# An *AARS1* variant identified to cause adult-onset leukoencephalopathy with neuroaxonal spheroids and pigmented glia

**DOI:** 10.1186/s40035-023-00353-1

**Published:** 2023-04-28

**Authors:** Jingying Wu, Taotao Liu, Benyan Zhang, Chang Liu, Xinghua Luan, Li Cao

**Affiliations:** 1 Department of Neurology, Shanghai Sixth People’s Hospital Affiliated to Shanghai Jiao Tong University School of Medicine, Shanghai, 200233 China; 2 Department of Neurology, The First Hospital Affiliated to Anhui University of Science & Technology, Huainan, 235099 China; 3 Department of Pathology, Ruijin Hospital Affiliated to Shanghai Jiao Tong University School of Medicine, Shanghai, 200025 China; 4 Department of Ophthalmology, Shanghai Sixth People’s Hospital Affiliated to Shanghai Jiao Tong University School of Medicine, Shanghai, 200233 China

Adult-onset leukoencephalopathy with spheroids and pigmented glia (ALSP) is a genetic disease characterized by progressive cognitive, movement and neuropsychiatric disorders, bilateral periventricular white matter hyperintensity in fluid attenuated inversion recovery (FLAIR) and diffuse weighted imaging (DWI) sequences, and axonal spheroids and pigmented microglia by brain biopsy. Heterozygous variants in *CSF1R* (colony stimulating factor 1 receptor) were firstly associated with ALSP (*CSF1R*-ALSP) [1]. Later, variants in *AARS2* (encoding alanyl-transfer tRNA synthetase 2) were found pathogenic for autosomal recessive ALSP patients (*AARS2*-ALSP) [2]. However, there is still a group of patients negative for mutations in both genes. Here, we report an autosomal-recessive ALSP family associated with *AARS1* mutation.

The patient from a consanguineous family suffered from difficulties in walking since age 23. At age 25, he was wheelchair-dependent with progressive dysarthria, blurred vision and cognitive decline. Physical examination showed marasmus (1.73 m height, 40 kg weight, body mass index 13.36), hypertonia, hyperreflexia and decreased muscle strength. Scores for Mini-mental State Examination and Montreal Cognitive Assessment were 12/30 and 3/30, respectively. Neurophysiological examinations revealed axonal impairment in the left superficial peroneal sensory nerve and motor fibers, and myelin damage in distal motor fibers of upper limbs. His uncorrected best vision was 4.0 and FC/20 cm; best corrected vision in the right eye was 4.2 and no improvement in the left eye through − 6.0D correction binocularly. Wide-angle fundus photography showed slender vessels in both eyes. Optical coherence tomography showed decreased thickness above the  retinal nerve fiber layer in the left eye and the outer nuclear layer of the left retina (Additional file 1: Fig. S1). No abnormality was revealed by electroencephalogram. Blood tests indicated hypoalbuminemia (26 g/l). His parents and younger brother had no neurological symptoms, as confirmed by electromyogram and magnetic resonance imaging (MRI).

Similar to patients with *CSF1R*-ALSP and *AARS2*-ALSP from our ALSP cohort, brain MRI of the patient revealed predominant symmetric hyperintensity of periventricular white matter and the corpus callosum in FLAIR (Fig. 1c-1) and DWI (Fig. 1d-1) sequences. Cortical atrophy was marked in T1 sequence (Fig. 1a-1, b-1). The subcortical U-fibers, cerebellum and midbrain were spared. CT scan (Fig. 1e-1) revealed symmetric patchy calcifications in the basal ganglia. [^18^F]FDG-PET/CT (Fig. 1f-1) showed diffusive hypometabolism, especially in the temporal and occipital lobes. [^18^F]DPA714-PET/CT illustrated symmetric bindings in the thalamus and the midbrain (Fig. 1g-1), indicating extensive neuroinflammation [3].

**Fig. 1:**
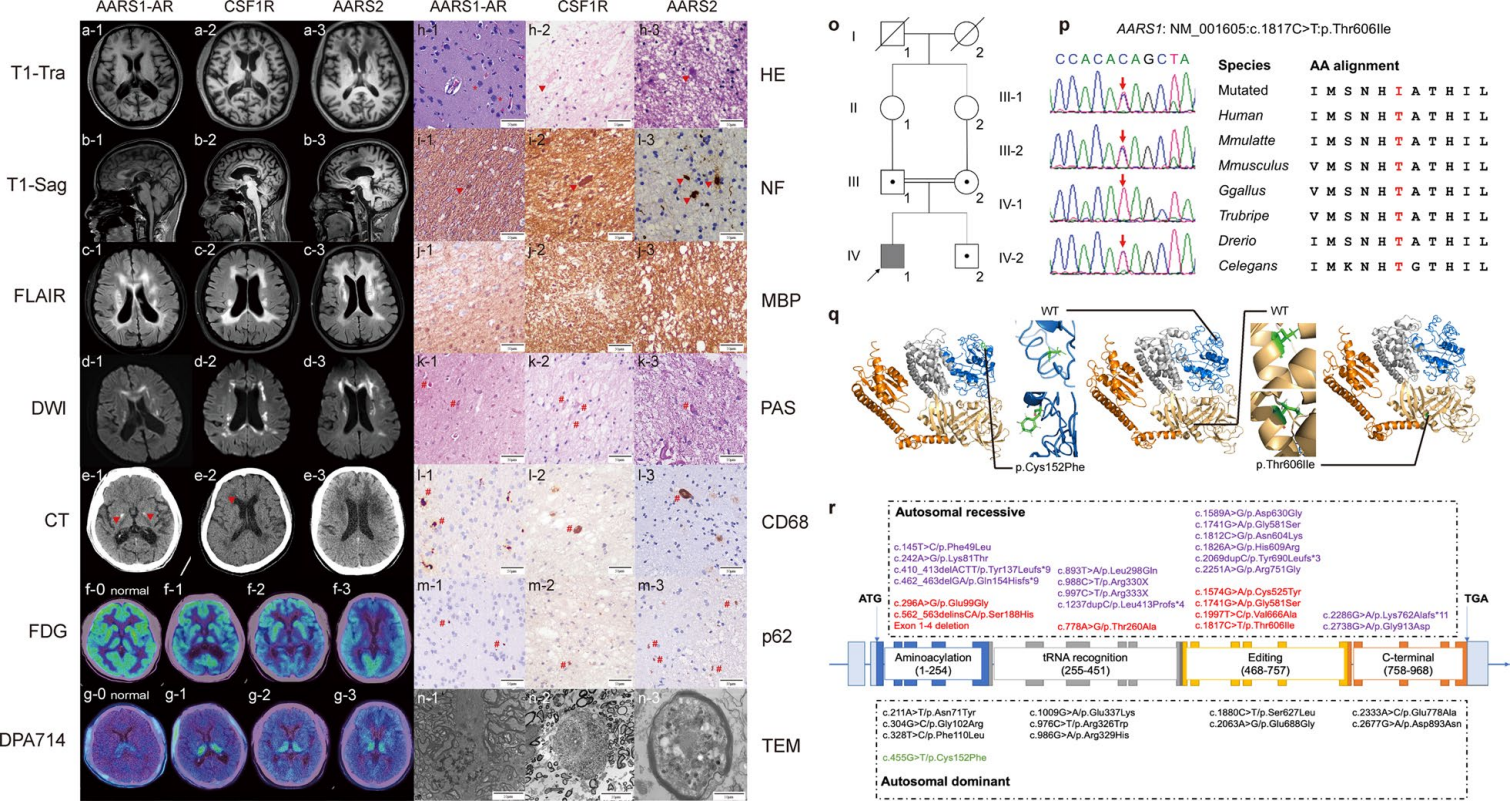
Radiological, pathological and genetic characteristics of *AARS1*-AR-ALSP, *CSF1R*-ALSP and *AARS2*-ALSP.** a–g** Sagittal and transverse view of neuroimaging. **h–n** Immunohistochemistry of brain biopsy. *, swollen neurons; red triangles, axonal spheroids; #, PAS-, CD68- or p62-positive microglia. **o–p** Genetic features of the family. **q** Predicted protein structures of  wild-type, p.Thr606Ile- and p.Cys152Phe-mutant AlaRS predicted by i-TASSER. Blue: aminoacylation domain; grey: tRNA recognition domain; light orange: editing domain; orange: C-terminal. **r** Diagram of AARS1 structure with reported mutations. Purple, DEE29-related variants; red, *AARS1*-AR-ALSP; black, CMT2N; green, *AARS1*-AD-ALSP

Biopsy showed mild loss of neurofilaments (Fig. 1h-1, i-1), swollen neurons (Fig. 1h-1), and sporadic spheroids (Fig. 1i-1). Myelin loss was prominent based on MBP staining (Fig. 1j-1). PAS-, CD68- and p62-positive microglia accumulated in the lesions (Fig. 1k-1, l-1, m-1). Electron microscopy (Fig. 1n-1) confirmed the loss of myelinated nerve fibers and existence of spheroids.

Based on these features, the proband was diagnosed as ALSP. However, trio analysis did not find any mutations related to *CSF1R* or *AARS2*, but revealed a homozygous nonsynonymous variant *AARS1*:NM_001605:exon14:c.1817C > T:p.Thr606Ile, as confirmed by Sanger sequencing. His parents and brother carried the same heterozygous variant (Fig. 1o, p). This variant was not found in 1000G Project, dbSNP, ESP6500 or gnomAD database, but predicted damaging by SIFT, PolyPhen-2 and MutationTaster. As the patient’s symptoms were highly consistent with previous cases of late-onset *AARS1*-related leukoencephalopathy [4], this variant was rated as “likely pathogenic” according to the American College of Medical Genetics and Genomics Standard. In silico prediction also revealed structural differences caused by the mutation (Fig. 1q).

*AARS1* gene encodes alanyl-tRNA synthetase (AlaRS), which catalyzes the aminoacylation of tRNA^Ala^ with alanine and diacylation of the incorrectly charged Ser-tRNA^Ala^ [5]. In aminoacylation and misaminoacylation assays, the AlaRS T606I variant showed deficient aminoacylation efficiency (Additional file 1: Fig. S2a) and higher mischarging rates (Additional file 1: Fig. S2b), supporting pathogenicity of this mutation.

ALSP was previously associated with *CSF1R* [1] and *AARS2 *[2] mutations, and one heterozygous variant in *AARS1* has been reported responsible for an ALSP family [6]. In this study, we associated a recessive *AARS1* variant with ALSP (*AARS1*-AR-ALSP).

ALSP patients associated with different genes have different characteristics (Additional file 1: Table S1). In summary, recessive subtypes have an earlier onset and are usually accompanied by ophthalmologic dysfunction and cerebellum or brainstem atrophy. Calcifications were only reported in *CSF1R*-ALSP and *AARS1*-AR-ALSP. *AARS1*-AR-ALSP patients  are also featured with posterior-predominant leukoencephalopathy, relatively mild pathological changes and neuroinflammation level, and hypoalbuminemia likely related to marasmus and malnutrition.

*AARS1* is also related to autosomal-dominant Charcot-Marie-Tooth disease type 2N (CMT2N) [7] and autosomal-recessive developmental and epileptic encephalopathy-29 (DEE29) [8]. Phenotype-genotype features are as follows (Fig. 1r; Additional file 1: Table S2).

(1) CMT2N-related variants are mostly located in tRNA recognition and aminoacylation domains, affecting aminoacylation activity [9], typified by the hotspot mutation c.986G > A/p.Arg329His.

(2) Variants of DEE29 are mostly located in the editing domain and the C-terminal, or cause truncated protein [8]. Patients with only one variant meeting the criteria have milder phenotype [10]. c.2251A > G/p.Arg751Gly and c.2738G > A/p.Gly913Asp [4] are the hotspot mutations.

(3) Variants of *AARS1*-AR-ALSP affect the editing domain, but none is located in the C-terminal or causes truncated protein.

Human AlaRS has higher mischarging activity compared to other aminoacyl-tRNA synthetases, explaining the importance of the editing domain [5]. The phenotypic differences also illustrated the potential critical role of the C-terminal domain [5], though its specific role remains unclear.

## Supplementary Information

**Additional file 1**: **Supplementary Methods.** **Fig. S1**. Ophthalmologic findings of the ALSP patient with *AARS1* mutation. **Fig. S2**. Aminoacylation and misaminoacylation kinetics assays of AlaRS T606I. **Table S1**. Clinical, imaging and pathological characteristics of *CSF1R*, *AARS2* and *AARS1*-related diseases. **Table S2**. Phenotype-genotype correlation in *AARS1*-related diseases.

## Data Availability

The datasets are available from the corresponding author on reasonable request.

## Abbreviations

AARS1: Alanyl-transfer tRNA synthetase 1 gene
AARS2: Alanyl-transfer tRNA synthetase 2 gene
AlaRS: Alanyl-tRNA synthetase
ALSP: Adult-onset leukoencephalopathy with neuroaxonal spheroids and pigmented glia
