# Supplementary material for: An AARS1 variant identified to cause adult-onset leukoencephalopathy with neuroaxonal spheroids and pigmented glia
Source: Transl Neurodegener. 2023 Apr 28;12:19. doi: 10.1186/s40035-023-00353-1 (PMC10142409; doi:10.1186/s40035-023-00353-1)
Supplement: Supplementary file 1 — Additional file 1: Supplementary Methods. Fig. S1. Ophthalmologic findings of the ALSP patient with AARS1 mutation. Fig. S2. Aminoacylation and misaminoacylation kinetics assays of AlaRS T606I. Table S1. Clinical, imaging and pathological characteristics of CSF1R, AARS2 and AARS1-related diseases. Table S2. Phenotype-genotype correlation in AARS1-related diseases. [file 40035_2023_353_MOESM1_ESM.pdf]

Additional file 1.

Pathological examination

Biopsied specimens were taken from cortex and periventricular white matter of the patient. Staining of formalin-fixed paraffin embedded sections included hematoxylin & eosin (H&E) and Schiff reagent (PAS). Immunohistochemistry was performed for myelin basic protein (abcam, #ab218011), CD68 (DAKO, #M087629), p62 (Biotechnology, #sc-28359) and neurofilament protein (NF, Cell Signaling Technology, #2836).

Plasmid construction, expression and protein purification

The construct expressing wild type human AlaRS was kindly provided by Dr. Litao Sun (Sun Yat-sen University). *Escherichia coli* AlaRS (*Ec*AlaRS) gene was inserted between NdeI and EcoRI sites of pET28. Gene mutagenesis was performed according to the protocol provided with the KOD-Plus Mutagenesis Kit. All constructs were transformed into *E. coli* BL21 (DE3) cells for gene expression, which was induced with a final concentration of 100 μM IPTG at 22°C for 10 h. Protein purification was performed as previously described [1].

tRNA transcription

Four complementary and overlapping oligonucleotides encoding the T7 promoter, the human cytoplasmic $tRNA^{Ala}$(AGC) gene, and its complementary chain were chemically synthesized, phosphorylated by T4 polynucleotide kinase, hybridized, and ligated by T4 DNA ligase into pTrc99b between EcoRI and PstI. tRNA transcripts were obtained by *in vitro* T7 RNA polymerase transcription as described previously [2].

Aminoacylation and misaminoacylation assays

Aminoacylation of $tRNA^{Ala}$(AGC) was carried out in a reaction mixture containing 50 mM Tris–HCl, pH 7.5, 20 mM KCl, 10 mM $MgCl_2$, 2 mM DTT, 4 mM ATP, 30 μM [^{14}C] Ala, 5 μM $tRNA^{Ala}$(AGC) with 200 nM AlaRS or T606I mutant. Every 2 minutes, reaction aliquots were removed to Whatman filter pads and subsequently processed in a similar procedure with previous report [2].

Misaminoacylation assay was carried out in a reaction mixture similar with aminoacylation reaction except 10 μM $tRNA^{Ala}$(AGC) and 2 μM AlaRS or T606I mutant used. A control was performed with *Ec*AlaRS C666A, which is a editing-defective tRNA synthetase [3]. Every 4 minutes, reaction aliquots were added to Whatman filter pads and subsequently processed in a similar procedure as above.

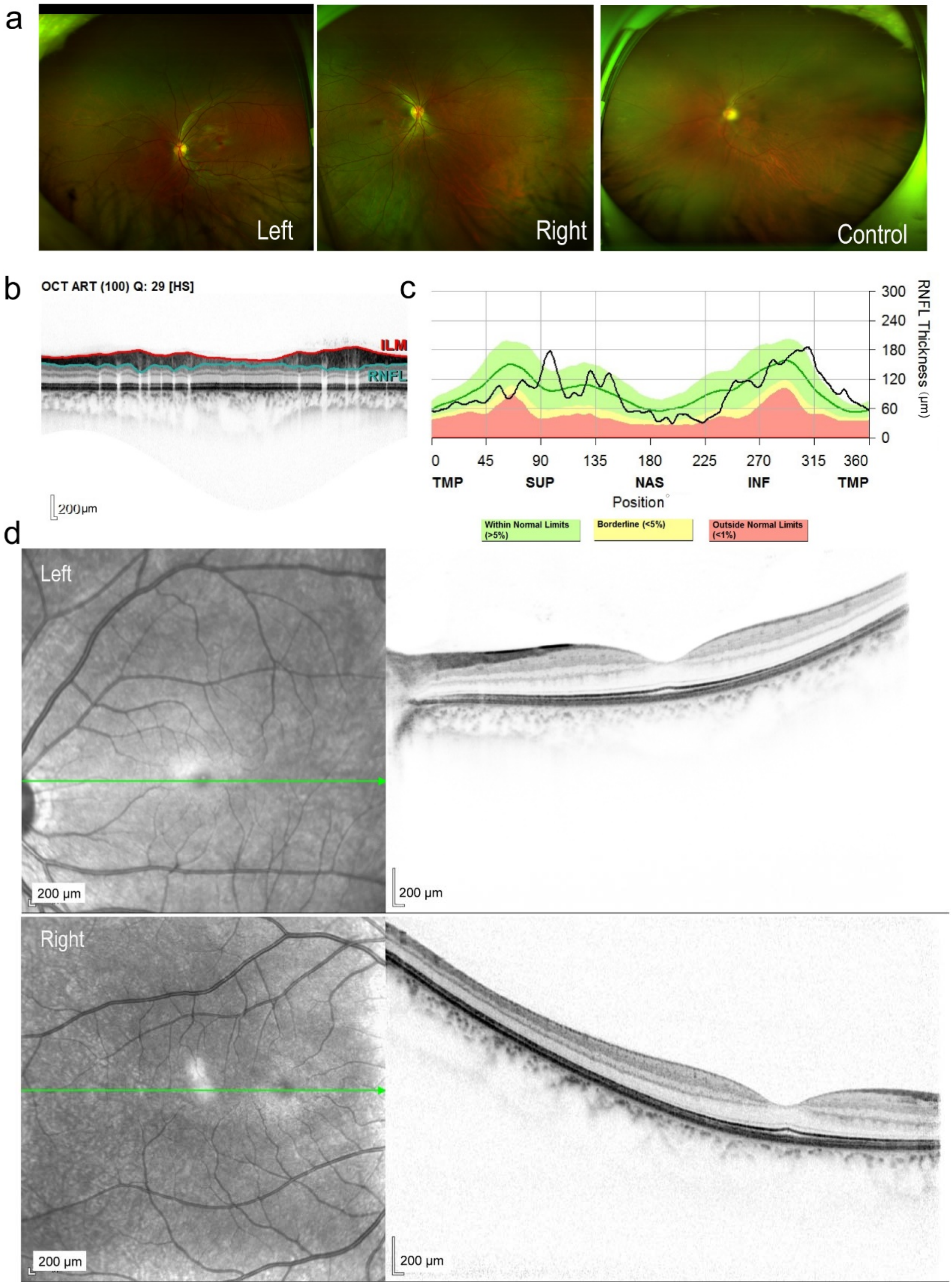

Figure S1 Ophthalmologic findings of the ALSP patient with *AARS1* mutation. Compared to his brother as control, Wide-angle fundus photography showing slender vessels in both eyes (**a**), and OCT showing thinner thickness above the RNFL layer in the left eye (**b**, **c**) and the outer nuclear layer of the left retina (**d**).

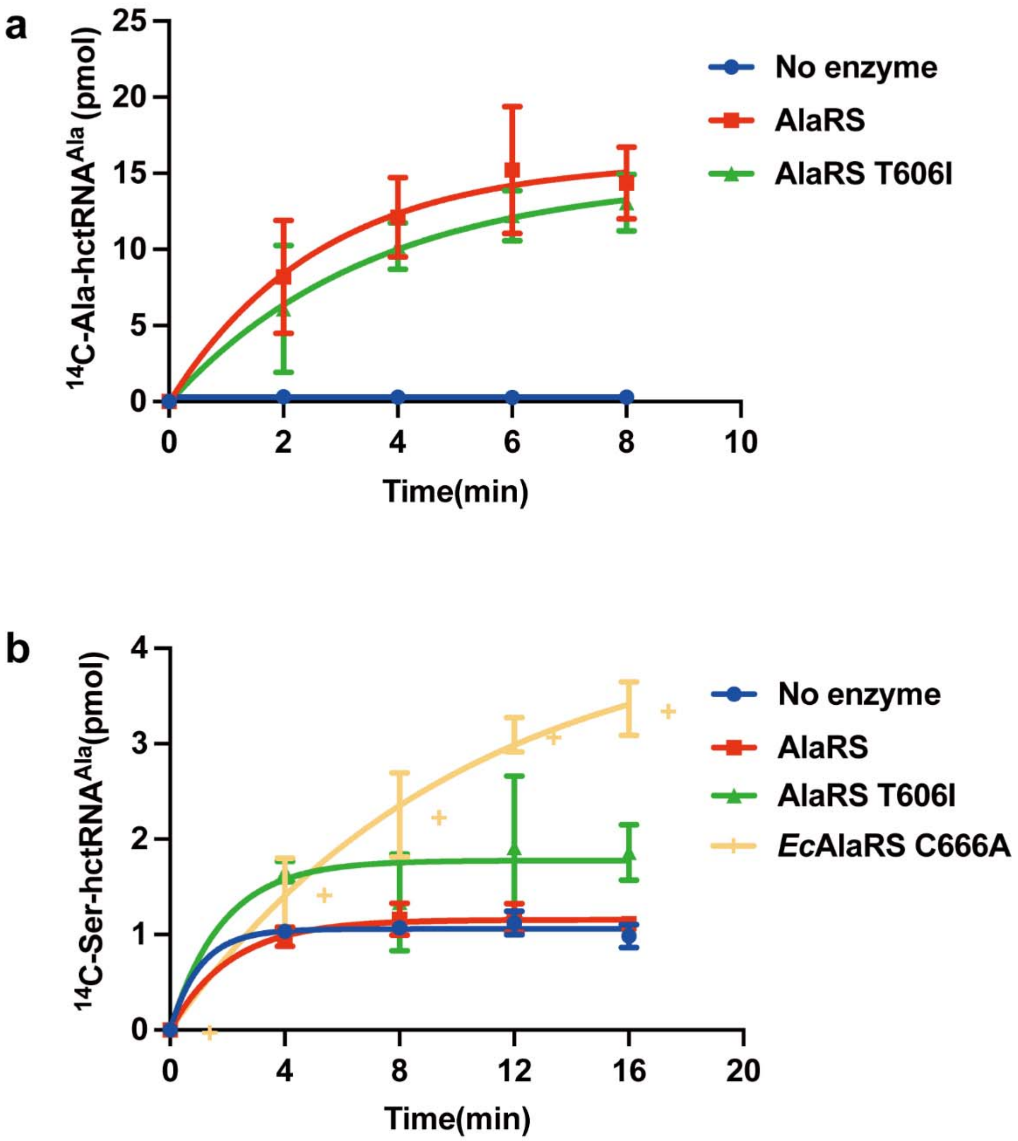

Figure S2 Aminoacylation and misaminoacylation kinetics assays of AlaRS T606I. (**a**) Aminoacylation of tRNA^{Ala} by alanine in the presence of AlaRS T606I and AlaRS. (**b**) Misaminoacylation of tRNA^{Ala} by serine in the presence of AlaRS T606I, AlaRS and *Ec*AlaRS C666A. In both graphs, the data represent the averages of three independent experiments and the corresponding standard deviations.

Table S1 Clinical, imaging and pathological characteristics of *CSF1R*-, *AARS2*- and *AARS1*-related diseases

		CSF1R-ALSP[4]	*AARS2*-ALSP[5-7]	*AARS1*-related diseases			
				AARS1-related leukoencephalopathy			AARS1-CMT[8-16]
				AARS1-DEE29[17-22]	AARS1-AR-ALSP[21]	AARS1-AD-ALSP[23]	
Inheritance pattern		Autosomal dominant (incomplete penetrance)	Autosomal recessive	Autosomal recessive	Autosomal recessive	Autosomal dominant	Autosomal dominant
Mean age of onset (range)[a]		43Y (18Y-78Y)	23Y (2M-45Y)	1M (0M-4M)	13Y (2Y-27Y)	40Y (19Y-55Y)	26Y (birth-60Y)
Initial symptoms		Gait disturbance, cognitive decline, psychiatric disorders	Mostly cognitive decline, ataxia and/or psychiatric disorders, some with spasticity and nystagmus	Microcephaly, development delay, hyperexcitability	Decline in mobility, speech and cognition	Cognitive decline, anxiety or parkinsonism	Mostly bilateral lower limbs weakness, or pain, cramps or numbness of distal lower limbs
Predominant Symptoms		Cognitive decline, parkinsonism, psychiatric disorders such as depression and anxiety, dysphonia, dysphagia	Cognitive decline, spasticity, cerebellar symptoms, psychiatric symptoms, dysphonia, dysphagia, ovarian failure in female	Microcephaly, development delay, spastic tetraparalysis, refractory myoclonic epilepsy	Cognitive decline, spastic tetraparalysis, dysphonia, dysphagia, seizures, blurred vision, no microcephaly	Cognitive decline, parkinsonism, psychiatric disorders	Weakness, amyotrophy and/or deformity of lower limbs, gradually developing into upper limbs, deafness in a few cases
Predominant Signs		Pyramidal signs such as spasticity, hyperreflexia and positive pathologic reflex, pseudobulbar palsy, ataxia	Pyramidal signs, ataxia, pseudobulbar palsy, nystagmus	Spasticity, hyperreflexia, hypotonia, extrapyramidal symptoms	Spasticity, hypotonia, hyperreflexia, ophthalmologic dysfunction	Pyramidal signs	Hypotonia, reflex loss and/or sensory loss in lower limbs or all limbs
Imaging	MRI	Bilateral white matter rarefaction in T1 and hyperintensity in T2/T2-FLAIR and DWI, punctate to confluently, involving periventricular regions, corpus callosum, pyramidal tracts, thin corpus callosum, moderate to severe cortical atrophy	Bilateral white matter rarefaction in T1 and hyperintensity in T2/T2-FLAIR and DWI, punctate to confluently, involving periventricular regions, corpus callosum, pyramidal tracts, cerebellum and brainstem, mild atrophy, thin corpus corpus	Severe atrophy involving cerebral grey matter and white matter, brainstem, cerebellum and posterior fossa, universal hypomyelination involving cerebrum as well as cerebellum and brainstem sometimes	Bilateral posterior prodominant leukoencephalopathy and cortical atrophy, not involving U-fibres, with thin corpus corpus and atrophy of frontal white matter, cerebellum and corticospinal tracts	Bilateral hypomyelination in periventricular regions, corpus callosum and pyramidal tracts, thin corpus callosum, moderate to severe cortical atrophy	Normal reported in two cases
	CT	Spotty white matter calcifications mainly in frontal and periventricular areas	No calcification	Calcification in cerebellar white matter and dentate nuclei in one case	Cerebral and/or cerebellar calcifications	No calcification	NA[b]

	PET	[18F]FDG-PET: Hypometabolism in frontoparietal areas [18F]DPA714-PET: Increased uptake in thalamus and midbrain	[18F]FDG-PET: Hypometabolism in frontoparietal areas [18F]DPA714-PET: dramatically increased uptake in frontoparietal cortex, thalamus and midbrain	NA[b]	[18F]FDG-PET: Hypometabolism in posterior areas [18F]DPA714-PET: mildly increased uptake in frontoparietal cortex, thalamus and midbrain	NA[b]	NA[b]
Pathology		Myelin and axon loss, axonal spheroids, pigmented CD68+ microglia	Myelin and axon loss, axonal spheroids, pigmented CD68+ microglia	NA[b]	Myelin and axon loss, axonal spheroids, pigmented CD68+ microglia, swollen neurons	Myeline and axon loss, axonal spheroids, pigmented CD68+ microglia	Myelopathy and axonopathy
Electrophy-siology	EMG	Mostly normal	Mostly normal	NA[b]	Peripheral neuropathy	NA[b]	Normal to severe sensorimotor axonal neuropathy
	EEG	Slight unusual EEG or epileptiform discharges in some patients	Slight unusual EEG or epileptiform discharges in some patients	Epileptiform discharges	Epileptiform discharge in some cases	NA[b]	NA[b]
Others features		Sex differences	Diffusely reduced cytochrome c oxidase staining in muscle,	Recurrent liver failure in one case	Hypoalbuminemia	-	-

Annotations:
[a] Y=years old, M=months old.
[b] NA=not available.

Table S2 Phenotype-genotype correlation in *AARS1*-related diseases

Family	Diagnosis	Mutation	Amino changes	Mutation type	Location[a]
F14[17]	DEE29	c.242A>C	p.Lys81Thr	Compound heterozygous	A
		c.2251A>G	p.Arg751Gly		E
F33[21]	DEE29	c.462_463delGA	p.Gln154Hisfs*9	Compound heterozygous	A~
		c.1741G>A	p.Gly581Ser		E
F32[21]	DEE29	c.410_413delACTT	p.Tyr137Leufs*9	Compound heterozygous	A~
		c.1589A>G	p.Asp530Gly		E
F30[21]	DEE29	c.2286G>A	p.Lys762Alafs*11	Homozygous	C~
F15[17]	DEE29	c.2251A>G	p.Arg751Gly	Homozygous	E
F22[19]	DEE29	c.1826A>G	p.His609Arg	Compound heterozygous	E
		c.2738G>A	p.Gly913Asp		C
F29[21]	DEE29	c.2251A>G	p.Arg751Gly	Compound heterozygous	E
		c.1812C>G	p.Asn604Lys		E
F16[18]	DEE29	c.2069dupC	p.Tyr690Leufs*3	Compound heterozygous	E~
		c.2738G>A	p.Gly913Asp		C
F24[20]	DEE29	c.893T>A	p.Leu298Gln	Compound heterozygous	R
		c.2251A>G	p.Arg751Gly		E
F35[22]	DEE29	c.1237dupC	p.Leu413Profs*4	Compound heterozygous	R~
		c.145T>C	p.Phe49Leu		A
F31[21]	DEE29	c.988C>T	p.Arg330X	Compound heterozygous	R~
		c.2738G>A	p.Gly913Asp		C
F34[21]	DEE29	c.997C>T	p.Arg333X	Compound heterozygous	R~
		c.2738G>A	p.Gly913Asp		C
F26[21]	*AARS1*-AR-ALSP	c.562_563delinsCA	p.Ser188His	Compound heterozygous	A
		c.1574G>A	p.Cys525Tyr		E
F25[21]	*AARS1*-AR-ALSP	c.296A>G	p.Glu99Gly	Compound heterozygous	A
		c.778A>G	p.Thr260Ala		R
F27[21]	*AARS1*-AR-ALSP	c.1741G>A	p.Gly581Ser	Homozygous	E
F28[21]	*AARS1*-AR-ALSP	c.1997T>C	p.Val666Ala	Compound heterozygous	E
		Exon1-4 deletion	Aminoacylation domain		A
Our case	*AARS1*-AR-ALSP	c.1817C>T	p.Thr606Ile	Homozygous	E
F21[23]	*AARS1*-AD-ALSP	c.455G>T	p.Cys152Phe	Heterozygous	A
F3[9]	CMT	c.211A>T	p.Asn71Tyr	Heterozygous	A
F13[13]	CMT	c.304G>C	p.Gly102Arg	Heterozygous	A
F17[14]	CMT	c.328T>C	p.Phe110Leu	Heterozygous	A
F6[11]	CMT	c.2677G>A	p.Asp893Asn	Heterozygous	C
F5[10]	CMT	c.2333A>C	p.Glu778Ala	Heterozygous	C
F12[12]	CMT	c.2063A>G	p.Glu688Gly	Heterozygous	E
F18[15]	CMT	c.1880C>T	p.Ser627Leu	Heterozygous	E
F20[15]	CMT	c.976C>T	p.Arg326Trp	Heterozygous	R
F1[8]	CMT	c.986G>A	p.Arg329His	Heterozygous	R
F2[8]	CMT	c.986G>A	p.Arg329His	Heterozygous	R
F4[10]	CMT	c.986G>A	p.Arg329His	Heterozygous	R
F7[12]	CMT	c.986G>A	p.Arg329His	Heterozygous	R
F8[12]	CMT	c.986G>A	p.Arg329His	Heterozygous	R
F9[12]	CMT	c.986G>A	p.Arg329His	Heterozygous	R
F10[12]	CMT	c.986G>A	p.Arg329His	Heterozygous	R
F11[12]	CMT	c.986G>A	p.Arg329His	Heterozygous	R
F23[16]	CMT	c.986G>A	p.Arg329His	Heterozygous	R
F19[15]	CMT	c.1009G>A	p.Glu337Lys	Heterozygous	R

Annotations:

[a]A = aminoacylation domain, R = tRNA recognition domain, E = editing domain, C = C-terminal domain, ~ = truncated protein.

References:

1. Zhou X-L, Zhu B, Wang E-D. The CP2 Domain of Leucyl-tRNA Synthetase Is Crucial for Amino Acid Activation and Post-transfer Editing. Journal of Biological Chemistry. 2008;283(52):36608-36616.
2. Mao XL, Li ZH, Huang MH, Wang JT, Zhou JB, Li QR*, et al.* Mutually exclusive substrate selection strategy by human m3C RNA transferases METTL2A and METTL6. Nucleic Acids Res. 2021;49(14):8309-8323.
3. Pasman Z, Robey-Bond S, Mirando AC, Smith GJ, Lague A, Francklyn CS. Substrate specificity and catalysis by the editing active site of Alanyl-tRNA synthetase from Escherichia coli. Biochemistry. 2011;50(9):1474-1482.
4. Papapetropoulos S, Pontius A, Finger E, Karrenbauer V, Lynch DS, Brennan M*, et al.* Adult-Onset Leukoencephalopathy With Axonal Spheroids and Pigmented Glia: Review of Clinical Manifestations as Foundations for Therapeutic Development. Front Neurol. 2021;12:788168.
5. Parra SP, Heckers SH, Wilcox WR, McKnight CD, Jinnah HA. The emerging neurological spectrum of AARS2-associated disorders. Parkinsonism Relat Disord. 2021;93:50-54.
6. Uzun GA. Adult-onset leukodystrophy with homozygous AARS2 mutation located in the aminoacylation domain. Neurol India. 2019;67(3):871-872.
7. Carle G, Morin A, Noiray C, Roy-Joly P, Cohen L, Levy R*, et al.* Alanyl-tRNA Synthetase 2-Related Dementia with Selective Bilateral Frontal Cystic Leukoencephalopathy. J Clin Neurol. 2018;14(3):420-422.
8. Latour P, Thauvin-Robinet C, Baudelet-Méry C, Soichot P, Cusin V, Faivre L*, et al.* A major determinant for binding and aminoacylation of tRNA(Ala) in cytoplasmic Alanyl-tRNA synthetase is mutated in dominant axonal Charcot-Marie-Tooth disease. Am J Hum Genet. 2010;86(1):77-82.
9. Lin KP, Soong BW, Yang CC, Huang LW, Chang MH, Lee IH*, et al.* The mutational spectrum in a cohort of Charcot-Marie-Tooth disease type 2 among the Han Chinese in Taiwan. PLoS One. 2011;6(12):e29393.
10. McLaughlin HM, Sakaguchi R, Giblin W, Wilson TE, Biesecker L, Lupski JR*, et al.* A recurrent loss-of-function alanyl-tRNA synthetase (AARS) mutation in patients with Charcot-Marie-Tooth disease type 2N (CMT2N). Hum Mutat. 2012;33(1):244-253.
11. Zhao Z, Hashiguchi A, Hu J, Sakiyama Y, Okamoto Y, Tokunaga S*, et al.* Alanyl-tRNA synthetase mutation in a family with dominant distal hereditary motor neuropathy. Neurology. 2012;78(21):1644-1649.
12. Bansagi B, Antoniadi T, Burton-Jones S, Murphy SM, McHugh J, Alexander M*, et al.* Genotype/phenotype correlations in AARS-related neuropathy in a cohort of patients from the United Kingdom and Ireland. J Neurol. 2015;262(8):1899-1908.
13. Motley WW, Griffin LB, Mademan I, Baets J, De Vriendt E, De Jonghe P*, et al.* A novel AARS mutation in a family with dominant myeloneuropathy. Neurology. 2015;84(20):2040-2047.
14. Karakaya M, Storbeck M, Strathmann EA, Delle Vedove A, Holker I, Altmueller J*, et al.* Targeted sequencing with expanded gene profile enables high diagnostic yield in non-5q-spinal muscular atrophies. Hum Mutat. 2018;39(9):1284-1298.
15. Weterman MAJ, Kuo M, Kenter SB, Gordillo S, Karjosukarso DW, Takase R*, et al.* Hypermorphic and hypomorphic AARS alleles in patients with CMT2N expand clinical and molecular heterogeneities. Hum Mol Genet. 2018;27(23):4036-4050.
16. Lee AJ, Nam DE, Choi YJ, Nam SH, Choi BO, Chung KW. Alanyl-tRNA synthetase 1 (AARS1)

gene mutation in a family with intermediate Charcot-Marie-Tooth neuropathy. Genes Genomics. 2020;42(6):663-672.
17. Simons C, Griffin LB, Helman G, Golas G, Pizzino A, Bloom M, *et al.* Loss-of-function alanyl-tRNA synthetase mutations cause an autosomal-recessive early-onset epileptic encephalopathy with persistent myelination defect. Am J Hum Genet. 2015;96(4):675-681.
18. Nakayama T, Wu J, Galvin-Parton P, Weiss J, Andriola MR, Hill RS, *et al.* Deficient activity of alanyl-tRNA synthetase underlies an autosomal recessive syndrome of progressive microcephaly, hypomyelination, and epileptic encephalopathy. Hum Mutat. 2017;38(10):1348-1354.
19. Krey I, Krois-Neudenberger J, Hentschel J, Syrbe S, Polster T, Hanker B, *et al.* Genotype-phenotype correlation on 45 individuals with West syndrome. Eur J Paediatr Neurol. 2020;25:134-138.
20. Marten LM, Brinkert F, Smith DEC, Prokisch H, Hempel M, Santer R. Recurrent acute liver failure in alanyl-tRNA synthetase-1 (AARS1) deficiency. Mol Genet Metab Rep. 2020;25:100681.
21. Helman G, Mendes MI, Nicita F, Darbelli L, Sherbini O, Moore T, *et al.* Expanded phenotype of AARS1-related white matter disease. Genet Med. 2021.
22. Leidi A, Previtali R, Parazzini C, Raviglione F, Carelli S, Mendes MI, *et al.* Correspondence on "Expanded phenotype of AARS1-related white matter disease" by Helman et al. Genet Med. 2022.
23. Sundal C, Carmona S, Yhr M, Almström O, Ljungberg M, Hardy J, *et al.* An AARS variant as the likely cause of Swedish type hereditary diffuse leukoencephalopathy with spheroids. Acta Neuropathol Commun. 2019;7(1):188.
